# Valorization of Fruit and Vegetable Pomace: Development of Zinc-Enriched Nutraceutical

**DOI:** 10.3390/foods15071219

**Published:** 2026-04-03

**Authors:** Tatjana Šoštarić, Zorica Lopičić, Snežana Zlatanović, Ferenc T. Pastor, Mihal Djuris, Stanislava Gorjanović

**Affiliations:** 1 Institute for Technology of Nuclear and Other Mineral Raw Materials, 11000 Belgrade, Serbia; z.lopicic@itnms.ac.rs; 2 Institute of General and Physical Chemistry, Studentski Trg 12/V, 11158 Belgrade, Serbia; snezana.zlatanovic@gmail.com; 3 University of Belgrade, Faculty of Chemistry, Studentski Trg 12, 11000 Belgrade, Serbia; fpastor@chem.bg.ac.rs; 4 University of Belgrade, Institute of Chemistry, Technology and Metallurgy—National Institute of the Republic of Serbia, Njegoševa 12, 11000 Belgrade, Serbia; mihal.djuris@ihtm.bg.ac.rs

**Keywords:** apple, beetroot, pomace, fluid-bed granulation, nutraceutical, zinc, vitamin C

## Abstract

Zinc deficiency is recognized as a global public health concern, affecting populations of all ages. This study aims to develop zinc supplements (nutraceuticals) based on by-products of the fruit and vegetable processing industry. Dehydrated apple and beetroot pomace powders were enriched with vitamin C and zinc via fluid-bed wet granulation, producing granules with substantially improved flowability (Carr’s index reduced by up to 45%, Hausner ratio by up to 25%, while the bulk and tapped density were reduced by up to 25% and 40%, respectively). Microbiological and long-term storage stability was demonstrated by low water activity (*aw*) (≤0.3), moisture content (<10%), and glass transition temperatures (T_g_ = 29–34 °C) that were well above standard storage conditions. The formulated nutraceuticals exhibited stronger antioxidant activity compared to the starting powders, as well as significant anti-hyperglycemic activity. Furthermore, the enhanced bioaccessibility of zinc was confirmed upon *in vitro* digestion of granulated samples, using atomic absorption spectrometry and differential pulse voltammetry. The present findings demonstrate that apple and beetroot pomaces can be successfully valorized as sustainable and functional matrices for zinc enrichment, being free of gluten, artificial sweeteners, colorants, preservatives, anti-caking agents, and anti-nutritional factors such as phytic acid.

## 1. Introduction

Zinc deficiency is a significant global public health concern. Approximately two billion individuals worldwide are at risk, primarily due to limited access to zinc-rich foods and dietary patterns dominated by cereals, nuts, and seeds, which contain high levels of phytates [1]. These anti-nutritional compounds chelate zinc and other micronutrients, reducing their bioavailability and inhibiting digestive enzymes. Globally, zinc deficiency is associated with an estimated 116,000 deaths annually, ranking as the second leading cause of mortality attributable to micronutrient deficiencies, following vitamin A deficiency [2].

Zinc is an essential trace element involved in a wide range of physiological processes, including neurocognitive function, digestion and metabolism, immune competence, endocrine regulation, and gene expression. Consequently, an inadequate zinc status leads to diverse and often severe health impairments. Notably, evidence emerging during the COVID-19 pandemic further highlighted the immunoprotective and therapeutic relevance of zinc, often in conjunction with vitamin C [3].

Type II diabetes is the most prevalent endocrine disorder globally and is characterized by chronic hyperglycemia accompanied by dysregulated lipid metabolism and impaired insulin function. Several mechanisms have been proposed to explain the pathogenesis of this disease, among which disturbances in zinc homeostasis and insufficient dietary fiber intake play significant roles. Zinc deficiency has been associated with an increased risk of developing Type II diabetes, as well as with the progression of diabetes-related complications [3]. In the early stages of the disease, insulin resistance is typically accompanied by compensatory hyperinsulinemia. Pancreatic β-cells secrete insulin together with zinc ions, and sustained insulin overproduction can lead to intracellular zinc depletion [4]. The excess zinc is eliminated primarily via the kidneys, which further reduces its availability for β-cell reuptake. Experimental studies have shown that, under *in vitro* conditions, zinc binds to insulin, enhancing its solubility within β-cells and improving its binding affinity to insulin receptors [5]. Collectively, these findings indicate that zinc plays an important role in glycemic regulation. In addition, zinc contributes to the modulation of lipid metabolism and may exert beneficial effects not only on diabetes, but also on obesity and inflammatory processes [6]. Zinc is an essential trace mineral that must be obtained from dietary sources or supplements, with an estimated adult requirement of 10–15 mg per day.

Alongside with growing interest in zinc and its health effects, the food industry faces increasing pressure to address environmental sustainability challenges. The fruit and vegetable processing sector generates large quantities of by-products, such as pomace, which are frequently underutilized or disposed of as waste. This waste contributes to environmental burdens, including increased landfill use, greenhouse gas emissions during decomposition, and the loss of valuable bioactive compounds that could otherwise be recovered. Consequently, valorizing these by-products aligns with global efforts to promote circular economy principles, reduce waste generation, and enhance resource efficiency within the agri-food industry.

Accordingly, there is increasing interest in utilizing juice-processing by-products such as pomaces as carriers for zinc. Previously, authors investigated apple and beetroot pomaces as valuable yet underutilized byproducts of the juice processing industry [7,8,9,10,11]. The selection of these two pomaces was motivated by several factors, including the ongoing annual increase in global apple production and the exceptional nutritional and bioactive profiles of both pomaces. In 2025, global apple production reached approximately 95 million tons, according to the FAO. Serbia is among the leading apple producers in South-East Europe, with an average annual output of around 420,000 tons. Apple pomace (AP), which makes up 10–30% of the fruit, is a highly perishable byproduct composed mainly of skin, pulp, seeds, and stalks. Its high moisture content renders it susceptible to rapid fermentation, posing challenges for proper disposal [7]. However, it contains phenolic compounds (catechins, procyanidins, phlorizin, phloretin glycosides, caffeic and chlorogenic acids, quercetin, and cyanidin glycosides), as well as dietary fibers (DF) such as soluble pectins, β-glucans, galactomannan gums, non-digestible oligosaccharides including inulin, and insoluble components like lignin, cellulose, and hemicelluloses. These constituents have been shown to exhibit antioxidative, cardioprotective, antidiabetic, gastroprotective, and antilipemic effects [8].

By contrast, beetroot production and consumption are smaller in scale, but the vegetable is increasingly popular due to their high levels of health-promoting compounds. Over the past decade, fruit juice consumption has declined, largely because of its high sugar content. This shift has prompted the beverage industry to focus on low- or no-calorie products, often incorporating vegetables like beetroot to reduce the sugar content [7]. Beetroot pomace (BR), which accounts for roughly 15–30% of the raw material after juice extraction, is typically discarded as waste. However, BR provides a rich nutritional profile, including carbohydrates, dietary fiber, protein, and a small proportion of fat, alongside essential minerals. It is also a valuable source of vitamins C, A, and B6; niacin; and folic acid, as well as bioactive compounds like betalains and polyphenols, which are responsible for their strong antioxidant and health-promoting effects [9]. Despite this, it is most often sent to landfills or, in limited cases, used as animal feed.

Therefore, both pomaces, which are rich in dietary fiber and free of phytic acid, demonstrate significant antioxidant activity, making them excellent candidates for mineral enrichment.

Powders obtained from pomaces through semi-industrial dehydration and subsequent grinding provide stable, nutritionally rich matrices for the incorporation of zinc and vitamin C via fluid-bed granulation. Such formulations are particularly relevant for individuals following vegan, vegetarian, or flexitarian diets; those adhering to gluten-free regimens; and individuals with prediabetes or diabetes.

Therefore, the aim of this study is to develop a product that is capable of replenishing zinc and vitamin C intake within the daily diet, while simultaneously providing high nutritional value. The intended product should be rich in dietary fiber and exhibit a pronounced antioxidant and anti-hyperglycemic effect. Crucially, both pomaces are entirely free of phytic acid, which is a key anti-nutritional factor that chelates zinc and reduces its bioavailability in cereal and legume-based matrices. This absence of phytic acid represents a significant compositional advantage over many other plant-based mineral carriers and was a primary criterion for their selection. Recent studies confirm that combining zinc with other compounds, particularly polysaccharides, enhances its bioavailability and amplifies its biological effects [12]. This evidence further supports the rationale for incorporating zinc into plant-based carriers that are rich in dietary fiber and polysaccharide structures, such as AP and BR.

## 2. Materials and Methods

### 2.1. Preparation of Zinc-Enriched Nutraceuticals

Preparation of Apple and Beetroot pomace powders

The starting materials were apple pomace (AP) and beetroot pomace (BR) obtained at an industrial scale (Fruvita, Smederevo, RS, Serbia), where no treatment other than pressing of thoroughly washed apples and beetroots was applied. Minimally processed AP and BR were collected aseptically immediately after pressing [8]. By applying a recently patented technological process [10], AP and BR were dehydrated at temperatures below 55 °C. The dehydrated AP and BR were grounded to a particle size below 300 µm and stored in multilayer paper sacks prior to analysis.

Fluid-bed Granulation of AP and BR

**Figure 1:**
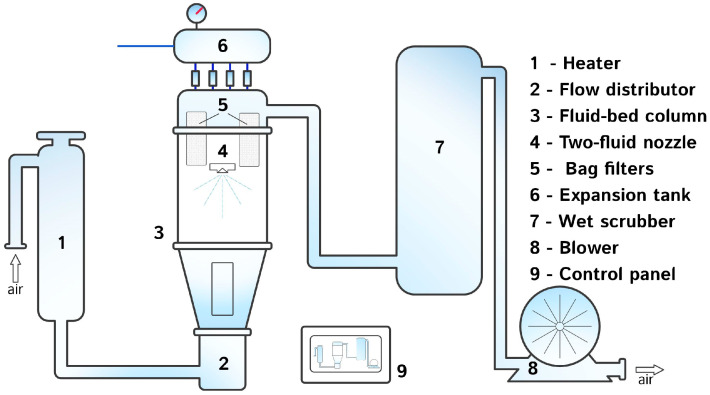
Schematic representation of the laboratory-scale fluid-bed granulator.

The process of wet granulation of starting excipients was performed in a custom-made fluid-bed device (Figure 1).

The stainless-steel fluid-bed granulator used in this study featured a conical bottom section that transitioned into a cylindrical expansion zone. The conical section had an internal diameter increasing from 125 mm at the air distributor to 215 mm at the cone–column interface, with a height of 260 mm (cone half-angle ≈ 10°), resulting in an expansion ratio of approximately 1.7. This geometry facilitated accelerated upward airflow and stable fluidization, promoting uniform particle circulation and controlled agglomeration. The frustum volume of the conical section was approximately 6 L. A centrally positioned top-spray two-fluid nozzle atomized the Zn solution, producing fine droplets that improved the wetting efficiency and increased the droplet–particle collision frequency. This configuration minimized spray losses and ensured homogeneous coating of the AP or BR powder.

The process of granulation procedure consisted of several steps:

- Sterilization of the starting sample (300 g of AP or BR) in an air stream at 75 °C for 10 min;
- Cooling of the sterilized sample to 48 °C;
- Top spraying of a binding solution on the samples in a fluidized bed (at binding fluid flow rate and air flow rate of 5 mL/min and 40 m^3^/h, respectively);
- Drying of granulated samples at 40 °C (under 30–40% lower air flow) until the moisture level was less than 5%.

After the drying phase, the samples were cooled to a room temperature and transferred into sterile polypropylene bags prior to further use. In the described wet granulation process, a water solution containing 3 mas% zinc (from ZnSO_4_), 16 mas% vitamin C and 0.7 mas% of polyvinylpyrrolidone (PVP) was used as the binding solution, in order to obtain AP-Zn and BR-Zn.

It is important to highlight the differences between standard granulation and fluid-bed granulation:

Principle: Standard granulation forms granules by adding a binder to a powder blend with mechanical mixing, while fluid-bed granulation fluidizes the powder with air while spraying a binder solution onto the particles.

Granule size control: Standard granulation offers limited control, while fluid-bed granulation allows precise control via spray rate, airflow, and drying time.

Uniformity: Standard granulation may produce heterogeneous granules, while fluid-bed granulation tends to provide more uniform coverage and homogeneity.

Process efficiency: Standard granulation is slower and often requires separate drying; fluid-bed granulation combines granulation and drying in a single step.

Energy usage: Fluid-bed granulation is generally considered more energy efficient, largely due to reduced processing time and integrated drying.

### 2.2. Determination of Physical Properties of AP and BR Before and After Granulation

The bulk density, tapped density, flowability, particle size distribution, and water activity (*aw*) of the powders were determined and compared before and after granulation, according to [13].

#### 2.2.1. Evaluation of the Flowability

The bulk and tapped densities were measured using a glass cylinder (23 mm diameter, 178 mm height, V = 73.95 mL). The tapped density was determined by mechanically tapping the cylinder 300–400 times until the powder reached maximum packing. All measurements were performed in triplicate, and the mean values were used to calculate the flowability parameters: the Carr Compressibility Index (CI) and Hausner ratio (H) [13]. Values are expressed as mean ± standard deviation (n = 3 independent measurements).

#### 2.2.2. Determination of Particle Size Distribution

Digital image analysis was used to determine the particle size distribution (PSD) by scanning powder samples before and after granulation. The particles were scanned with an HP Scanjet 300 scanner (registred in Böblingen, Germany, manufactured in Chongqing, China), and the images were stored at a resolution of 4800 dpi (pixel size 5.29 µm), which was sufficient to accurately capture particle boundaries and ensure reliable measurements. The scanned images were analyzed using ImageJ v.1.53g (University of Wisconsin–Madison, Madison, WI, USA) to determine the particle size, expressed as the projected area diameter (dA) [14]. The PSD was determined by image analysis of 1000–2000 randomly selected particles. Analysis of more than approximately 1000 particles did not significantly change the distribution (variation < 2%), indicating good statistical representativeness.

#### 2.2.3. Determination of Water Activity

Water activity (*aw*) of non-granulated and granulated materials were determined weekly by the *aw* meter Novasina LabSwift Bench-model Water Activity Meter (Neutec Group Inc., Farmingdale, NY, USA) at 25 ± 2 °C. For each sample, three independent experiments were performed in triplicate.

#### 2.2.4. Determination of Thermal Stability: Differential Scanning Calorimetry (DSC)

A differential scanning calorimeter (DSC Q1000, TA Instruments, New Castle, DE, USA) was employed to assess the thermal stability of samples before and after the granulation process. Approximately 5–7 mg of each sample (three independent replicates) was sealed in aluminum pans. Samples were first cooled from 20 to −90 °C, held isothermally for 5 min, and subsequently heated from −90 to 250 °C at a rate of 5 °C/min under a nitrogen purge (50 mL/min). Thermograms were processed using TA Advantage Universal Analysis 2000 software (version 4.5A). The glass transition was determined from the change in heat capacity (ΔC_p_) and expressed as onset (T_g,on_), midpoint (T_g_), and endset (T_g,end_) temperatures. The DSC instrument equipped with Advanced T_zero_™ technology enables the direct determination of heat capacity in a single scan.

### 2.3. Determination of Chemical Properties of AP and BR Before and After Granulation

#### 2.3.1. Determination of Proximate Composition

The ash and moisture contents of both samples were measured following the ASTM standard procedures: ASTM E871-82 (2013) [15] for moisture, ASTM E1755-01 (2015) [16] for ash and ASTM E872 (2019) [17] for volatile matter determination.

#### 2.3.2. Determination of Total Polyphenolic Content and Antioxidant Activity

##### Preparation of Samples by *In Vitro* Digestion

The standardized INFOGEST protocol [18] was used to assess the *in vitro* digestibility of the powders before and after granulation. This method reproduces the sequential oral, gastric, and intestinal phases of digestion. All procedures were performed in triplicate. A blank solution containing all reagents except the granulated samples or control (ZnSO_4_) was included to account for any potential background contamination.

Oral phase simulation: Powder samples before and after granulation (1 g) were placed in 50 mL conical tubes and mixed with 2.5 mL of simulated salivary fluid, 0.5 mL of α-amylase (1500 U/mL), 25 µL of 0.3 M CaCl_2_, and 7.475 mL of distilled water. The pH was adjusted to 7.0, and the mixture was incubated at 37 °C for 2 min to simulate oral digestion.

Gastric phase simulation: Following the oral phase, 5 mL of gastric electrolyte solution, prepared from stock solutions of KCl, KH_2_PO_4_, NaHCO_3_, NaCl, MgCl_2_, and (NH_4_)_2_CO_3_ according to the INFOGEST protocol, was added to 10 mL of the oral bolus. Subsequently, 1.6 mL of pepsin solution (25,000 U/mL) and 5 µL of 0.3 M CaCl_2_ were added. The pH was adjusted to 3.0 using 1 M HCl, and distilled water was added to reach a final volume of 20 mL. The mixture was then incubated at 37 °C for 2 h under constant agitation at 250 rpm.

Intestinal phase simulation: After gastric digestion, 11 mL of intestinal electrolyte solution—containing bile salts and pancreatin and prepared according to the INFOGEST protocol—was added to the gastric chyme. The pH was adjusted to 7.0 using 1 M NaOH, and distilled water was added to bring the final volume to 40 mL. The mixture was incubated at 37 °C for 2 h under agitation. Following digestion, the samples were centrifuged, and the supernatant was collected for subsequent analyses of bioaccessible antioxidant activity while both the supernatant and precipitate were used for the determination of the zinc content.

##### Determination of Total Polyphenolic Content

The Folin–Ciocalteu (FC) assay was used to quantify the total phenolic content in digested powders and granulated powders. Appropriately diluted samples (0.5 mL) were mixed with 2.5 mL of FC reagent (diluted 11-fold), and allowed to react in the dark for 5 min. Subsequently, 2 mL of 7.5% sodium carbonate solution was added, and the mixture was shaken and then left to react for 2 h in the dark. Absorbance was measured at 760 nm, using distilled water as a blank. All measurements were performed in triplicate, and the results were expressed as milligrams of gallic acid equivalents per gram of sample (mg GAE/g) [19].

##### Determination of Antiradical Activity Using the DPPH Assay

The antiradical activity of the samples against the DPPH radical was evaluated using a modified version of the Blois method [20]. Three different concentrations of diluted samples (0.2 mL each) were mixed with 2.8 mL of a 0.1 mM DPPH solution prepared in a 2:1 (*v*/*v*) mixture of ethanol and 0.1 M acetate buffer at pH 4.3. The mixture was shaken and incubated in the dark for 1 h prior to measuring the absorbance at 525 nm. The results were expressed as millimoles of Trolox equivalents per gram of sample (mM TE/g).

##### Determination of Total Reducing Power Using the FRAP Assay

The FRAP assay was conducted by following a previously described protocol with minor modifications [21]. The FRAP reagent was prepared by mixing 0.3 M acetate buffer (pH 3.6), 10 mM 2,4,6-Tri(2-pyridyl)-s-triazine (TPTZ) dissolved in 40 mM HCl, and 20 mM FeCl_3_ in a volume ratio of 10:1:1, respectively. Aliquots of diluted samples (0.1 mL) were combined with 0.3 mL of distilled water and 3 mL of FRAP reagent. The mixtures were incubated in the dark for 40 min, after which the absorbance was measured at 593 nm. The results were expressed as millimoles of Trolox equivalents per gram of digested sample (mM TE/g).

##### Relative Antioxidant Capacity Index (RACI)

Central tendency measures were applied to compare the antioxidant activity of powders before and after granulation, assessed by different assays, the total phenolic content (TPC), ferric reducing antioxidant power (FRAP) and radical scavenging activity (DPPH). Digested samples were ranked based on the mean and standard deviation of values obtained from each assay. Given the differences in units and scales across these antioxidant assays, the raw data from each assay were transformed into standardized scores. These scores are dimensionless values calculated by subtracting the mean from the raw data and dividing them by the standard deviation. The standardized scores for each sample across all assays were then averaged to yield a single unitless value known as the Relative Antioxidant Capacity Index (RACI). RACI provides a unified metric that integrates results from multiple antioxidant assays with differing units, allowing for direct comparison without variance distortion. It serves as a robust indicator of the overall antioxidant activity across diverse analytical techniques [22,23].

#### 2.3.3. Determination of Zinc Content in Samples Subjected to *In Vitro* Digestion

##### Determination of Zinc Content by Atomic Absorption Spectroscopy

The zinc content in solid samples was determined by atomic absorption spectroscopy- AAS (PerkinElmer PinAAcle 900T (PerkinElmer, Inc., Shelton, CT, USA). A known amount of each sample (2.0 g) was digested with concentrated HNO_3_ and HClO_4_ until complete dissolution. The digest was cooled and HCl was added. The zinc concentrations were measured from the solution by using AAS with a zinc hollow cathode lamp at 213.9 nm, and the sample concentrations were calculated from a calibration curve prepared with Zn standard solutions.

##### Determination of Zinc Content by Differential Pulse Voltammetry

The supernatant obtained upon the centrifugation of digested samples for 15 min at 6800 RPM was used for Zn determination without further treatment. After drying at 60 °C to constant mass, the precipitate was homogenized in porcelain mortal. A total of 200 mg of the homogenized dry precipitate was transferred in a microtube, thoroughly mixed with 2 mL of the ammonia buffer ([NH_4_Cl] = 1M, [NH_3_] = 2M, pH = 9.3) and left overnight. Samples were centrifuged at 13,400 rpm for 10 min, and the supernatant was analyzed.

In the polarographic cell, 8 mL H_2_O, 2 mL ammonia buffer ([NH_4_Cl] = 1M, [NH_3_] = 2M, pH = 9.3) and 100 µL of analyzed supernatant were added. The solution was deaerated with pure (5.0) nitrogen for 5 min. Differential pulse voltammograms were scanned between 1.0 and 1.4 V, with a pulse amplitude of 50 mV, and a scan rate of 10 mV/s. Metrohm multimode electrode pro in SMDE mode was used as a working, glassy carbon electrode as an auxiliary and a 3.0 M KCl Ag/AgCl electrode as a reference. The Autolab PGSTAT101 (Metrohm, Herisau, Switzerland) was connected to a Metrohm 663 VA stand over the Autolab IME663 interface.

#### 2.3.4. *In Vitro* Anti-Hyperglycemic Activity Assay

The anti-hyperglycemic activity of the initial powders and granulated products subjected to digestion according to the Infogest procedure was examined using α-glucosidase inhibitory potential (AHgA), conducted according to Tumbas Šaponjac et al. [24]. Acarbose was used as a control.

### 2.4. Statistics

Statistical analysis was performed using XLSTAT (version 2014.5.03, Addinsoft, New York, USA), an add-in for Microsoft Excel. Results are presented as mean ± standard deviation (SD) of three independent measurements (n = 3). The statistical significance was determined by one-way ANOVA followed by Tukey’s post hoc test, with differences considered to be significant at *p* < 0.05.

## 3. Results and Discussion

### 3.1. Preparation of Zinc-Enriched Nutraceutical

Two types of zinc enriched nutraceuticals (AP-Zn and BR-Zn) were obtained at pilot scale level, using the apple and beetroot pomace powders as a zinc carrier. The concentrations of ZnSO_4_, vitamin C and PVP (binder) were optimized to obtain the desired characteristics of the final products by wet fluid-bed granulation. Both materials underwent characterization, and the results are summarized below.

### 3.2. Physical Properties

#### 3.2.1. Flowability

The physical properties of granulates, and thus the quality of the granulated product, were determined based on the comparison of bulk and tapped densities, Carr’s index of compressibility (*CI*) and Hausner ratio (*H*) (Table 1). As seen, the values of the bulk and tapped densities decreased for both powders after the granulation process. Carr’s Index and Hausner ratio were used to evaluate powder flowability: a lower value of *CI* indicates a better flow and the value of *H* being closer to 1 indicates a better powder flow, while *H* > 1.4 indicates a poor flow [25]. As can be seen from Table 1, there is a significant decrease in the *CI* and *H* values compared to in the starting materials. This indicates that the flowability of the starting powders is significantly improved after the granulation process, ensuring easier handling, transport, and packaging of the obtained granulated materials. Future studies will focus on formulating hard capsules and tablets to ensure uniform granulate distribution and to evaluate critical properties—flowability, compressibility, and compactness—that are essential for both efficient capsule filling and direct compression of uncoated tablets.

As shown in Table 1, Zn enrichment significantly affected the physical properties of the powders (one-way ANOVA, *p* < 0.05). The decrease in the Carr index and Hausner ratio values indicates the improved flowability of the granulated products compared to the initial pomace powders.

#### 3.2.2. Particle Size Distribution

The degree of granulation was also determined by the comparison of distribution size curves for the initial powder particles and granulated product. The results presented in Table 1 show successful granulation of the pomace powders: the initial particle range increased from 20 < *d_p_* < 460 to 25 < *d_p_* < 625 μm for AP-Zn, and to 25 < *d_p_* < 725 μm for BR-Zn; in the same time, the average particle diameter increased from 90 to 225 μm and from 90 to 195 μm for AP and BR, respectively. This widening of the particle size range, along with the increase in the average particle diameter, clearly indicates the formation of stable agglomerates during the fluid-bed wet granulation process. These changes confirm the efficient particle growth and successful conversion of the initial fine powders into coarser, free-flowing granules.

#### 3.2.3. Water Activity

The low water activities (*aw*) of 0.3 and 0.39 of the starting powders AP and BR, enabled efficient grinding of dehydrated pomace and ensured good stability and storability of the obtained powders. As indicated in Table 2, a lower water activity enhanced the stability of the granulated powders. The lower water activity (*aw* = 0.23) further supports the product stability, as values below 0.3 effectively inhibit microbial growth and reduce chemical and enzymatic degradation [26].

#### 3.2.4. Thermal Characteristics of Granulated Products

Thermal analysis additionally confirmed the stability of the granulated powders (AP-Zn and BR-Zn) at typical storage temperatures (Table 2). The glass transition temperatures (T_g_) of both granule types (AP-Zn and BR-Zn) were higher than the typical storage temperatures, indicating that the materials remain in a glassy state during storage. This condition minimizes the molecular mobility and diffusion processes, thereby contributing to the physical and biochemical stability of the granules. Similar findings were reported by [27], who emphasized that maintaining a storage temperature below T_g_ enhances the stability of low-moisture food systems.

ANOVA revealed significant differences among the samples for T_g,on_, T_g_, T_g,end_, and water activity (*p* < 0.05), while no significant differences were observed for ΔC_p_ (*p* > 0.05). Low water activity coupled with a glass transition temperature above the storage temperature suggests that AP-Zn and BR-Zn granules remain stable at room temperature, maintaining their structural and functional integrity during storage.

### 3.3. Chemical Properties

#### 3.3.1. Proximate Composition

The organic composition of the apple and beetroot powders was reported previously [7,11,28]. Proximate analysis of AP and BR powders demonstrated a significantly reduced total sugar content—defined as the sum of glucose, fructose, and sucrose—relative to whole apple and beetroot powders, together with markedly higher concentrations of soluble and insoluble dietary fiber (AP ~ 40 g/100 g dw, BR ~ 30 g/100 g dw), while their protein content differs slightly (AP ~ 4 g/100 g dw and BR ~ 14 g/100 g dw) [7]. As a result, the AP and BR powders exhibited a lower carbohydrate-to-fiber ratio, a recognized marker of enhanced nutritional quality.

As presented in Table 3, changes in the ash content were observed among the samples. It increased after granulation, from 1.72% in the apple powder-based carrier (AP) to 4.58% in AP-Zn, and from 6.43% in the beet powder-based carrier (BR) to 8.78% in BR-Zn, indicating that zinc was successfully incorporated into both granules. In addition, the moisture content decreased after granulation, from 7.81% in AP to 5.96% in AP-Zn, and from 8.15% in BR to 4.38% in BR-Zn, showing that the granulated materials are drier than the original powders, which contributes to the stability of the final products. ANOVA analysis revealed significant differences among the samples for the moisture, volatile matter, and ash content.

#### 3.3.2. Total Polyphenolic Content and Antioxidant Activity

The antioxidant (AO) activity of the powders, both before and after the granulation process, was determined following *in vitro* digestion. The digested samples were evaluated for their ability to reduce Fe(III) and to scavenge free radicals using the FRAP (Ferric Reducing Antioxidant Power) and DPPH (2,2-diphenyl-1-picrylhydrazyl radical) assays. Additionally, the total polyphenol content (TPC) was measured using the Folin–Ciocalteu (FC) method, which is not strictly specific to phenolic compounds and essentially measures the total reducing capacity. Thus, it can be considered as an additional AO test that contributes to a more reliable assessment of the overall AO activity of the analyzed samples. The values presented in Table 4 confirmed a significant increase in the AO activity of granulated samples in comparison to starting ones.

##### Evaluation of AO Activity Based on Relative Antioxidant Capacity Index (RACI)

The Relative Antioxidant Capacity Index (RACI) is frequently used to enable the comparison of AO activity determined by parallel application of multiple assays. RACI represents the mean value of standardized results transformed from raw data generated by various assays. As a unitless numerical scale, RACI aligns consistently with various assays, representing a valid tool for evaluating AO activity. Although RACI is a relative index and does not represent a specific antioxidant property, it provides an adequate comparison of antioxidant activity among various samples such as food items and can therefore be used to assess the antioxidant activity.

**Figure 2:**
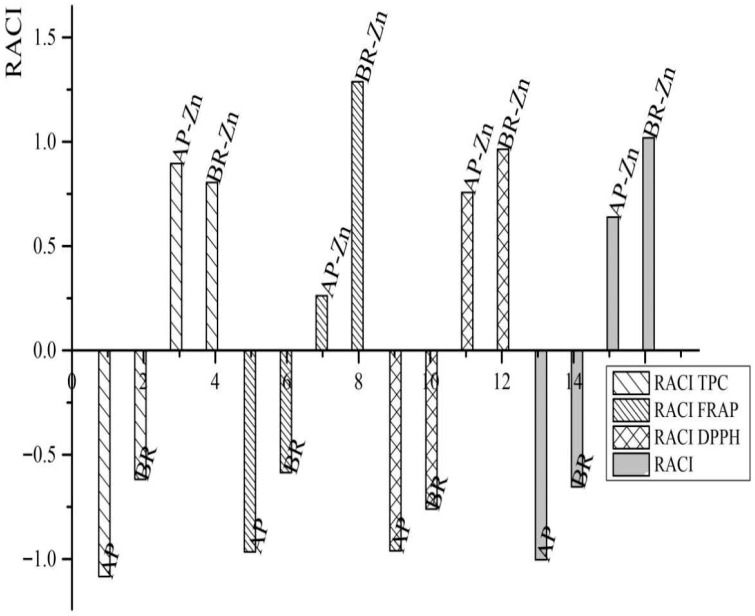
Comparative view of the initial pomace powders (AP and BR) and granulated products (AP-Zn and BR-Zn). RACI calculated by taking into account DPPH, FRAP and TPC, separately and collectively.

According to the RACI (Table 4 and Figure 2) conducted based on AO activity determined using DPPH, FRAP and TPC, the total AO activity ranking was 1.02 for BR-Zn and 0.64 for AP-Zn, while BR and AP showed values of −0.65 and −1.00, respectively. These results confirmed that BR-Zn and AP-Zn possess significantly higher AO activity compared to BR and AP. These results can be associated with the addition of vitamin C, which was added to promote zinc absorption. The effect is related to zinc ascorbate, the zinc salt of ascorbic acid, which forms a chelated compound through the interaction of Zn^2+^ ions with vitamin C [29]. This complex is widely used as a dietary supplement because ascorbic acid improves zinc absorption and facilitates its cellular uptake [30]. Therefore, the antioxidant activity of the granulated samples reflects the additive contributions of vitamin C and phenolic compounds that are naturally present in the starting materials. The results presented in Table 4 and Figure 2 confirm not only the enhancement of the granulates’ AO, but also demonstrate that the granulation process had no negative impact on it. Also, the results presented in Table 4 and Figure 2, along with the obtained results of the proximate composition, validated the efficiency of the technological process in preserving biomolecules in their active form and highlighted the potential application of the granules as nutraceuticals. ANOVA indicated significant differences among the samples in TPC, FRAP, and DPPH antioxidant activities (*p* < 0.05). The Zn-containing samples (AP-Zn and BR-Zn) showed a higher antioxidant capacity, as reflected in higher RACI values.

#### 3.3.3. Zinc Bioaccessibility: *In Vitro* Assessment

Nutrient bioavailability is the fraction of intake that is absorbed and utilized by the body, occurring in three phases: intestinal absorption, systemic distribution, and cellular utilization [31]. Zinc bioavailability is difficult to assess due to the lack of specific biomarkers, so absorption is often measured as bioaccessibility, the portion of a nutrient released from food and available for absorption. Furthermore, the bioaccessibility and bioavailability of nutrients, including minerals, can be measured with *in vitro* or in vivo methods. In zinc research, *in vitro* simulated digestion is the most common and is usually referred to as bioaccessibility. In vivo studies often use stable isotope techniques to measure the true bioavailability [32].

Beyond the added vitamin C, known to enhance bioaccessibility, the intrinsic nutritional properties of the starting materials, namely the absence of phytates and their high fiber and polysaccharide content [7,28,29], have been shown to substantially improve zinc bioavailability [12]. Notably, recent research indicates that zinc sulfate exhibits the highest bioaccessibility when consumed with a high-fiber diet [32]. This prompted the authors to investigate whether a tailored formulation could improve zinc bioaccessibility, even from zinc sulfate, the most cost-effective supplement.

*In vitro* digestion of the powders before and after granulation was conducted, following the INFOGEST protocol to assess zinc bioaccessibility and its potential intestinal absorption. The zinc content in the digested samples was determined using atomic absorption spectroscopy (PerkinElmer PinAAcle 900T (PerkinElmer, Inc., Shelton, CT, USA)) and polarographic analysis.

The results, presented in Table 5, are expressed as the percentage of zinc bioaccessibility, relative to the initial concentration. Depending on the analytical method applied, zinc bioaccessibility ranged from 35 to 40% for AP-Zn and 45 to 54% for BR-Zn. In comparison, Rock [33] reported a zinc bioaccesibility of 24% only from zinc sulphate, as determined using the INFOGEST protocol, which further decreased to 8% with an increasing phytic acid to zinc molar ratio. In addition, Tokarczyk [32], who investigated the bioaccessibility of various zinc salts (including zinc sulfate) in relation to dietary composition (i.e., fiber content) demonstrated that the bioaccessibility increases with a higher proportion of dietary fiber (from 2.00% to 8.75%), but only in the case of zinc sulfate, while a decrease was observed for other inorganic zinc salts. The notably higher values obtained in this study suggest that this effect arises from the advantageous properties of the starting materials, the absence of phytic acid, the high dietary fiber content, and the presence of added vitamin C, all of which are known to enhance zinc bioaccessibility.

Our findings support the effectiveness of this strategy. Our future work will include the zinc bioavailability by in vivo study on animals exposed to a single optimal dose of AP-Zn and BR-Zn, calculated on the basis of zinc bioaccessibility according to the *in vitro* study.

#### 3.3.4. *In Vitro* Anti-Hyperglycemic Activity

Regular apple consumption improves insulin resistance and lowers the risk of type 2 diabetes [34]. Beetroot consumption also exhibits antidiabetic effects by inhibiting carbohydrate digesting enzymes and modulating the gut microbiota, while enzyme and thermal treatment additionally increase its antidiabetic activity [35]. During the juice production process, the reduction in sugar content enhances the functional value of apple or beetroot pulp, as the majority of sugars are transferred into the juice, while bioactive compounds with antidiabetogenic properties remain largely preserved in the pulp. Through careful processing by dehydration pomace, stabilization was achieved. The potential of the obtained AP and BR with an extended shelf life as a carrier of microelements has not yet been sufficiently investigated. A long-term animal study considering the glycemic status of C57BL/6J mice exposed to high-fat and sucrose supplemented with AP, as well as glucose tolerance determined by an oral glucose tolerance test, demonstrated its prominent anti-diabetic effect. The animal groups that were fed a standard and high fat and sucrose diet supplemented with a low amount of AP had significantly suppressed BWG: 60.7% and 39.2%, respectively. Both BWG and the feed efficiency ratio (weight gain/food intake) (FER) significantly differ between diets with and without AP. The diet with AP addition resulted in 2.6 and 1.7 reduced FER in comparison to the control groups [8]. Similar results (unpublished) were observed in long-term supplementation of animal diets with BR, using the identical experiment design, which means that both AP and BR represent adequate material as Zn carriers. The vitamin C and Zn are expected to potentiate the antidiabetic effect of AP and BR demonstrated.

The anti-hyperglycemic activities of the samples were evaluated to determine their potential use as nutraceuticals, not only for zinc supplementation but also for improving glycemic control. Hyperglycemia is a condition characterized by abnormally elevated plasma glucose levels, which, when accompanied by insulin resistance, can lead to the development of type II diabetes. The growing number of individuals with diabetes, along with the adverse side effects associated with synthetic antidiabetic drugs, has intensified the search for alternative or adjunctive therapies with minimal undesirable effects. One such agent is acarbose, which delays the rise in postprandial glucose levels by inhibiting the enzymes α-amylase and α-glucosidase, both of which play key roles in the digestion of complex carbohydrates [36]. The enzyme α-glucosidase, located in the intestinal tract, participates in the final stage of carbohydrate digestion by catalyzing the breakdown of starch and disaccharides into glucose [37]. The current research is increasingly focused on identifying new α-amylase and α-glucosidase inhibitors from natural sources in order to reduce the side effects associated with the inhibitors that are currently used for hyperglycemia management. The results present additional evidence demonstrating that AP-Zn and BR-Zn exhibit anti-hyperglycemic activity; specifically, they attenuate the rise in postprandial glucose levels by inhibiting α-glucosidase. Anti-hyperglycemic activity was assessed by following the method described by [24], where acarbose served as the reference inhibitor. Within the tested concentration range, all samples inhibited α-glucosidase *in vitro*, confirming their anti-hyperglycemic potential (Table 6).

The test results indicate that anti-hyperglycemic activity increases with the concentration of all samples. The literature suggests that polyphenolic compounds can interact with proteins and inhibit the activity of various enzymes, including α-amylase and α-glucosidase [36]. A comparison of the IC_50_ values of the tested samples shows that all exhibit significant anti-hyperglycemic activity. Notably, approximately half the concentration of BR-Zn and AP-Zn (29.4 and 29.9 mg/mL, respectively) is sufficient to reduce α-glucosidase activity by 50%, compared to the starting BR and AP samples. ANOVA showed significant differences in acarbose inhibitory activity among the samples at 50 and 100 mg/mL, as well as in IC_50_ values (*p* < 0.05), while no significant differences were observed at 25 mg/mL. The zinc-containing samples (AP-Zn and BR-Zn) showed stronger inhibitory activity and lower IC_50_ values compared to AP and BR. The α-glucosidase inhibitory activity is likely attributable to the phenolic compounds and dietary fiber, both well-known inhibitors of carbohydrate-hydrolyzing enzymes. According to [36], the α-glucosidase inhibition assay showed that blueberries and blackberries inhibited at least 50% of α-glucosidase activity within 60 min under the applied experimental conditions, which is consistent with the results of the present study for AP and BR. Also, the concentrated grape pomace extracts exposed pronounced inhibitory activity on both α-amylase and α-glucosidase, close to acarbose [38]. These findings suggest that natural α-glucosidase inhibitors derived from plant sources such as agro-industrial residues, as well as the fiber present [39], may offer an effective strategy for controlling hyperglycemia, especially combined with zinc and vitamin C. Based on the *in vitro* findings, an animal study will be designed and conducted, followed by an in vivo clinical study involving patients with insulin resistance.

Given the high content of fiber and bioactive polyphenolics of the AP-Zn and BR-Zn granules, along with the demonstrated biological activity, these materials are suitable for the development of nutraceutical formulations, including capsules, tablets, and sachets, aimed at preventing metabolic disorders and addressing micronutrient deficiencies such as zinc insufficiency. It can supply the recommended daily intake of zinc and vitamin C, while also providing additional health benefits associated with their substantial antioxidant capacity and pronounced anti-hyperglycemic activities. Taking into account that the recommended daily intake of zinc for adults is 10–15 mg, the desired formulation (e.g., tablets) can be developed in such a manner to ensure that the final product provides 15 mg of zinc, corresponding to the upper limit of the recommended dose. The preliminary results indicate that the average mass of the uncoated tablets prepared by direct compression of AP-Zn was 437 ± 9 mg. Due to the good flowability, compressibility, and compactibility of the granules, it is possible to produce tablets by direct compression without the addition of any excipients. For the tablets manufactured using flat-faced tooling with a diameter of #13 mm, the average zinc content was 9.8 mg per tablet. To achieve a lower or higher zinc content per tablet, tools with a smaller or larger diameter would need to be used. For a zinc content of 15 mg per tablet, either a larger granule mass per tablet (~670 mg for the specified tooling) or larger-diameter tooling, such as #15 mm, would be required. It is important to emphasize that the process allows for straightforward adjustment to a lower zinc content, enabling appropriate dosing for children. Based on its composition, the developed nutraceutical may also be suitable for pediatric use. In addition, the obtained granules can be incorporated into functional food products, either in powder form or as an additive, to enhance their nutritional and biological value.

Considering that this product is manufactured through a sustainable process and does not contain substances that are harmful to human health, the resulting supplement can be classified as a highly desirable option due to its favorable composition and its positive impact on both human well-being and the environment. With the growing trend toward healthier lifestyles, consumer interest in such products continues to rise, as an increasing number of individuals actively read labels and seek to verify the quality and transparency of a product’s ingredients.

## 4. Conclusions

The results confirm that wet fluid-bed granulation simultaneously improved the physicochemical properties of the apple (AP) and beetroot (BR) powders and enabled the direct formation of zinc-enriched granules, thereby demonstrating their suitability as zinc carriers. Reduced values of Carr’s index (up to 45%), Hausner ratio (up to 25%), bulk density (up to 25%), and tapped density (up to 40%), relative to the starting material, directly indicating the improved flowability of the obtained materials. Low water activity (below 0.3) and moisture content (below 10%), together with glass transition temperatures (T_g_) of 29–34 °C, which exceed storage temperatures, suggest good physical stability and the potential for prolonged shelf life.

The pronounced antioxidant and anti-hyperglycemic activities of AP and BR were further enhanced by fluid-bed granulation with the addition of vitamin C, which, as expected, increased the antioxidant activity while supporting zinc absorption. The total antioxidant activity, evaluated after *in vitro* digestion, demonstrated a significant increase compared to the starting materials, as confirmed by the RACI (BR-Zn: 1.02; AP-Zn: 0.64). The anti-hyperglycemic activity was similarly enhanced (IC_50_ = 29.9 mg/mL for AP-Zn and 29.4 mg/mL for BR-Zn), with the observed effects being attributable to the combined contributions of vitamin C and pomace-derived phenolic compounds.

These findings highlight the potential of the obtained granulates for zinc supplementation, which are particularly suited to vegans, vegetarians, flexitarians, and individuals with prediabetes or diabetes who are on gluten-free diets. Additionally, this work provides a foundation for further investigation of pomace powders as carriers of various bioactive compounds and micronutrients. Future research will focus on *in vivo* bioavailability studies, extended stability testing under real storage conditions, and enrichment with additional micronutrients to develop broader-spectrum nutraceutical formulations based on pomace matrices.

## Figures and Tables

**Table 1:** Comparison of physical properties of the initial pomace powders and granulated products.

Parameters	AP	AP-Zn	BR	BR-Zn
*ρ_bulk_* (g/mL)	0.49 ± 0.01 ^a^	0.36 ± 0.01 ^c^	0.42 ± 0.01 ^b^	0.32 ± 0.01 ^d^
*ρ_tapped_* (g/mL)	0.69 ± 0.01 ^a^	0.45 ± 0.01 ^b^	0.68 ± 0.01 ^a^	0.41 ± 0.01 ^b^
*CI*	29.67 ± 1.77 ^a^	18.45 ± 0.85 ^c^	38.20 ± 1.40 ^b^	21.51 ± 1.12 ^c^
*H*	1.42 ± 0.03 ^a^	1.23 ± 0.02 ^c^	1.62 ± 0.04 ^b^	1.27 ± 0.02 ^c^
particle size range (μm)	20 < *d_p_* ^1^ < 460	25 < *d_p_* ^1^ < 625	20 < *d_p_* ^1^ < 460	25 < *d_p_* ^1^ < 725
*d*_50_ ^2^ (μm)	90	225	90	195

AP—apple pomace; BR—beetroot pomace; AP-Zn—granulated apple pomace loaded with Zn; BR-Zn—granulated beetroot pomace loaded with Zn; ^1^—particle diameter; and ^2^—average particle diameter. Values are presented as mean ± standard deviation (n = 3 independent measurements). Values with the same superscript letter are not significantly different, while those with different letters (a–d) differ significantly (*p* < 0.05), as determined by one-way ANOVA followed by Tukey’s post hoc test.

**Table 2:** Thermodynamical parameters obtained from DSC curves and water activity (*aw*).

Parameters		AP	AP-Zn	BR	BR-Zn
Glass transition temperature	T_g,on_ (°C)	29.3 ± 0.9 ^a^	26.9 ± 0.9 ^b^	34 ± 1.0 ^c^	33.9 ± 0.7 ^c^
	T_g_ (°C)	31.8 ± 1.2 ^b^	28.8 ± 1.1 ^b^	35 ± 2.0 ^a^	36 ± 1.0 ^a^
	T_g,end_ (°C)	44.9 ± 1.9 ^a^	31.3 ± 1.8 ^c^	41 ± 1.0 ^ab^	38 ± 3.0 ^b^
	∆C_p_ (J/(g°C)	3.1 ± 1.0 ^a^	1.35 ± 0.21 ^a^	2.1 ± 0.8 ^a^	1.8 ± 0.5 ^a^
Water activity, *aw*		0.30 ± 0.01 ^b^	0.23 ± 0.01 ^c^	0.39 ± 0.01 ^a^	0.23 ± 0.01 ^c^

AP—apple pomace; BR—beetroot pomace; AP-Zn—granulated apple pomace loaded with Zn; and BR-Zn—granulated beetroot pomace loaded with Zn. Values are presented as mean ± standard deviation (n = 3 independent measurements). Values with the same superscript letter are not significantly different, while those with different letters (a–c) differ significantly (*p* < 0.05), as determined by one-way ANOVA followed by Tukey’s post hoc test.

**Table 3:** Proximate composition of starting and granulated products.

Parameters	AP	AP-Zn	BR	BR-Zn
Moisture (%)	7.81 ± 0.13 ^a^	5.96 ± 0.11 ^b^	8.15 ± 0.08 ^c^	4.38 ± 0.09 ^d^
Volatile matter (%)	79.61 ± 1.5 ^a^	78.12 ± 1.25 ^a^	75.56 ± 1.64 ^b^	74.22 ± 1.43 ^b^
Ash (%)	1.72 ± 0.03 ^a^	4.58 ± 0.05 ^b^	6.43 ± 0.06 ^c^	8.78 ± 0.05 ^d^

AP—apple pomace; BR—beetroot pomace; AP-Zn—granulated apple pomace loaded with Zn; and BR-Zn—granulated beetroot pomace loaded with Zn. Different superscript letters within the same row indicate statistically significant differences between samples (one-way ANOVA followed by Tukey’s post hoc test, *p* < 0.05).

**Table 4:** Comparison of AO activity of the initial pomace powders and granulated products, measured by DPPH, FRAP, and TPC assays, along with RACI values calculated for each assay separately and collectively.

	TPC mg GAE/g	RACI TPC	FRAP µmol TE/g	RACI FRAP	DPPH µmol TE/g	RACI DPPH	RACI
BR-Zn	43.6 ± 5.4 ^a^	0.80	361 ± 30 ^a^	1.29	496 ± 27 ^a^	0.96	1.02
AP-Zn	45.4 ± 3.9 ^a^	0.89	207 ± 8 ^b^	0.26	445 ± 63 ^a^	0.76	0.64
BR	15.1 ± 1.4 ^b^	−0.62	80 ± 2 ^c^	−0.59	72 ± 3 ^b^	−0.76	−0.65
AP	5.8 ± 5.4 ^c^	−1.08	23 ± 2 ^d^	−0.96	22 ± 2 ^b^	−0.96	−1.00

AP—apple pomace; BR—beetroot pomace; AP-Zn—granulated apple pomace loaded with Zn; BR-Zn—granulated beetroot pomace loaded with Zn. Different superscript letters within the same row indicate statistically significant differences between samples (one-way ANOVA followed by Tukey’s post hoc test, *p* < 0.05).

**Table 5:** Zinc bioaccessibility upon *in vitro* gastrointestinal digestion of the initial pomace powders and granulated products, determined by AAS and DPV, expressed as % of total Zn.

Method	AP-Zn (%)	BR-Zn (%)
AAS	35.3 ± 0.6	44.7 ± 0.7
DPV	40.1 ± 0.8	53.7 ± 0.9

AP-Zn—granulated apple pomace loaded with Zn; and BR-Zn—granulated beetroot pomace loaded with Zn.

**Table 6:** Anti-hyperglycemic activity of the initial pomace powders and granulated products.

	% of Inhibition			
Conc. (mg/mL)	25	50	100	IC_50_
BR	17.4 ± 2.2 ^a^	27.5 ± 2.2 ^b^	75.6 ± 3.4 ^c^	73.4 ± 2.8 ^a^
BR-Zn	23.8 ± 3.7 ^a^	40.3 ± 0.9 ^a^	95.4 ± 1.7 ^a^	29.4 ± 1.2 ^c^
AP	20.1 ± 1.0 ^a^	37.2 ± 2.4 ^a^	86.5 ± 3.9 ^b^	63.0 ± 3.1 ^b^
AP-Zn	22.3 ± 1.9 ^a^	38.6 ± 1.9 ^a^	96.6 ± 0.9 ^a^	29.9 ± 1.0 ^c^
Acarbose	-	-	-	0.002 ± 0.00

AP—apple pomace; BR—beetroot pomace; AP-Zn—granulated apple pomace loaded with Zn; BR-Zn—granulated beetroot pomace loaded with Zn. Different superscript letters within the same row indicate statistically significant differences between samples (one-way ANOVA followed by Tukey’s post hoc test, *p* < 0.05).

## Data Availability

The original contributions presented in this study are included in the article. Further inquiries can be directed to the corresponding authors.
